# Transcriptomic profiling of feline teeth highlights the role of matrix metalloproteinase 9 (MMP9) in tooth resorption

**DOI:** 10.1038/s41598-020-75998-3

**Published:** 2020-11-03

**Authors:** S. Lee, S. J. Bush, S. Thorne, N. Mawson, C. Farquharson, G. T. Bergkvist

**Affiliations:** 1 The Royal (Dick) School of Veterinary Studies and The Roslin Institute, The University of Edinburgh, Easter Bush Campus, Midlothian, EH25 9RG UK; 2 Nuffield Department of Medicine, University of Oxford, Oxford, OX3 7LF UK; 3 DentalVets, Apex House, Alderston Field, Haldane Avenue, Haddington, EH41 3NQ UK

**Keywords:** Cell biology, Computational biology and bioinformatics, Molecular biology, Diseases, Molecular medicine, Pathogenesis

## Abstract

Tooth resorption (TR) in domestic cats is a common and painful disease characterised by the loss of mineralised tissues from the tooth. Due to its progressive nature and unclear aetiology the only treatment currently available is to extract affected teeth. To gain insight into TR pathogenesis, we characterised the transcriptomic changes involved in feline TR by sequencing RNA extracted from 14 teeth (7 with and 7 without signs of resorption) collected from 11 cats. A paired comparison of teeth from the same cat with and without signs of resorption identified 1,732 differentially expressed genes, many of which were characteristic of osteoclast activity and differentiation, in particular matrix metalloproteinase 9 (MMP9). MMP9 expression was confirmed by qPCR and immunocytochemistry of odontoclasts located in TR lesions. A hydroxamate-based MMP9 inhibitor reduced both osteoclast formation and resorption activity while siRNA targeting MMP9 also inhibited osteoclast differentiation although had little effect on resorption activity. Overall, these results suggest that increased MMP9 expression is involved in the progress of TR pathogenesis and that MMP9 may be a potential therapeutic target in feline TR.

## Introduction

Tooth resorption (TR) of permanent teeth is the pathological loss of dental hard tissues and has been reported in many animals including humans, dogs, horses, domestic cats and wild cats^1–5^. Affecting multiple teeth, resorption is common in aged domestic cats but is rarer in other animals^6–8^. Destructive and progressive lesions usually initiate on the external surface of the coronal tooth root and extend into the cementum, enamel, dentine and in advanced stages, the pulp^7,9,10^. TR largely classifies into three Types (Type 1–3) by the Nomenclature Committee of the American Veterinary Dental College (AVDC). Type 1 is characterised by appearance of focal or multifocal lesions of the tooth with normal periodontal ligament and inflammatory changes while Type 2 presents resorption lesions with involvement of periodontal ligament and non-inflammatory replacement resorption^11^. Type 3 exhibits both features of Type 1 and Type 2. TR is caused by tooth-resorbing odontoclasts, cells functionally similar to bone-resorbing osteoclasts^7^. Both cell types are derived from circulating blood or bone marrow myeloid lineage progenitors which differentiate into mature multinucleated cells in their respective microenvironments^10,12–15^. The main physiological role of odontoclasts is to resorb hard tissues around deciduous teeth allowing tooth eruption to commence during tooth development and to resorb the deciduous teeth for permanent tooth replacement^16^. In some cats these cells become dysregulated and attack the permanent teeth later in life^9,17^. Due to recognition of this disease and investigations into the prevalence of TR between the 1990s and 2000s, the aetiology of TR has been widely studied^9,18^. The central axis of differentiation of osteoclasts and odontoclasts is the pathway comprising colony stimulating factor 1 (CSF-1) and the receptor activator of nuclear factor κ B (RANK), RANK ligand (RANKL), and osteoprotegerin (OPG), a decoy receptor, capturing RANKL^12,15^. Since the microenvironments of physiological and pathologic TR are different, there has been a focus on identifying co-stimulating factors in pathological conditions. The accumulated evidence suggests that the initiation and progression of TR is complex and multifactorial and is also mediated by local rather than systemic mechanisms. Many possible molecules and risk factors that directly or indirectly impact on the RANK/RANKL/OPG axis pathway have been postulated, including pro-inflammatory cytokines^19,20^, diet^21,22^, thyroid hormones^23^, parathyroid hormone^24,25^, parathyroid-related peptides^26^, and vitamin D and its metabolites^17,27^. However, the precise causes of TR in cats remain unknown.

Transcript identification and the quantification of gene expression have been distinct core activities in molecular biology ever since the discovery of RNA’s role as the key intermediate between the genome and the proteome. To our knowledge, no whole transcriptomic analysis of the feline tooth and dental microenvironment has been reported. We recently reported an optimised protocol for RNA extraction from cat teeth that produced RNA of acceptable quality for generation of cDNA libraries suitable for RNA-seq^28^. The aims of this study were firstly to perform RNA-seq analysis to compare the feline tooth transcriptome of normal and TR affected teeth. We then identified candidate genes related to feline odontoclastic regulation pathways and targeted them using RNA interference and inhibitors to further evaluate their role in odontoclast differentiation and odontoclast dentine resorption. The transcriptomic changes identified in TR teeth will help us further understand the pathogenesis of TR and help identify genes that can be considered potential therapeutic targets for the treatment of odontoclast dysregulation.

## Material and methods

### Tooth collection and phenotyping of TR

Teeth were collected from patients with full owner’s consent presented to the Hospital for Small Animals, The Royal (Dick) School of Veterinary Studies, The University of Edinburgh, UK. TR type and staging were confirmed by review of dental radiographs by veterinary dental specialists, S. Thorne and Dr Norman Johnston (MRCVS, RCVS, American & European Specialist in Veterinary Dentistry, DentalVets, Haddington). Further research samples including teeth, maxilla, mandibles and long bones were collected at post mortem from cats euthanized for a wide range of medical or ethical reasons, not related to tooth resorption, and donated to the school for research. Ethical approval for the study was granted by the R(D)SVS veterinary ethical review committee (VERC reference 05.13). All methods were carried out in accordance with the relevant guidelines and regulations.

### Tissue processing and RNA extraction

Samples including teeth, mandibles and maxilla were snap frozen in liquid nitrogen and stored at − 80 °C until RNA extraction. Information of samples are described in Table 1. Teeth were extracted from the alveolar sockets using dental equipment or using bone cutters while maintaining cold temperatures by working over dry ice. Tissue processing and RNA extraction protocols were optimised based on a guanidinium thiocyanate-phenol–chloroform extraction method and manufacturer’s instructions for the Qiagen RNeasy Mini Kit. This protocol has been previously documented^28^.

**Table 1:** Phenotyping of TR and selection of samples for RNA sequencing.

Sample ID	Extracted teeth	TR status	TR type	TR stage	Description of TR status	RIN^e^	RNA yield (mg)	Age	Sex
N14C12TR−	Right/left 3rd and 4th PM	−	N/A	N/A	No TR lesion	4.5	10.3	4	F
N28C15TR−	Right/left 3rd PM	−	N/A	N/A	No TR lesion	7.3	9.0	9	FN
N29C25TR−	Right/left 3rd PM	−	N/A	N/A	No TR lesion	6.7	8.0	10	FN
N19C21TR−	Left 4th PM	−	N/A	N/A	TR-tooth from TR + cat	5.3	8.2	16	FN
N21C27TR−	Right 3rd PM	−	N/A	N/A	Paired TR-tooth	4.1	8.4	7	FN
N23C30TR−	Left 1st M	−	N/A	N/A	Paired TR-tooth	4.4	9.0	10	FN
N25C32TR−	Left 4th PM	−	N/A	N/A	Paired TR-tooth	4.9	8.7	9	FN
N08C8TR+	Right/left 3rd PM	+	2	2	TR + tooth from TR + cat	5.2	5.8	> 5	F
N30C16TR+	Left 4th PM	+	2	2	TR+ tooth from TR + cat	4.7	9.1	5	FN
N27C23TR+	Right 4th PM	+	3	4	TR+ tooth from TR + cat	7.3	0.8	6	FN
N32C29TR+	Right 4th PM	+	3	4	TR+ tooth from TR + cat	7	9.7	7	FN
N22C27TR+	Right C	+	2	2	Paired TR + tooth	5.9	7.4	7	FN
N24C30TR+	Left 3rd PM	+	2	2	Paired TR + tooth	5.2	9.0	10	FN
N26C32TR+	Right 3rd PM	+	3	4	Paired TR + tooth	6.3	8.5	9	FN

RIN^e^: the RNA integrity number equivalent, N/A: not applicable, F: female, N: RNA sample number, C: cat number, FN: female neutered, RNA yield shows total yield. PM; premolar, M; molar, C; canine.

### cDNA library and RNA sequencing

Tested RNA samples were sent to our academic facility (Edinburgh Genomics, The University of Edinburgh, UK) for cDNA library production and RNA sequencing. Briefly, one microgram of RNA from each of the thirteen samples and 0.8 µg of RNA from one low quantity sample (N27C23TR+) were used to generate fourteen cDNA libraries using the Illumina TruSeq stranded mRNA sample preparation kit according to the manufacturer’s instructions (Illumina, San Diego, CA, USA). Paired-end sequencing was performed using the Hiseq 4000 system (Illumina). The low quality reads were filtered by Phred quality score (Q score 30) and 3′ adapter were trimmed with cutadapt (version 1.8.3). All the raw reads have been submitted to the European Nucleotide Archive (ENA) under accession PRJEB24183 (ENA, https://www.ebi.ac.uk/ena). Assessment of quality control of RNA and cDNA library are stated elsewhere^28^.

### Mapping to the feline reference genome and generation of read counts

Alignments to the *Felis catus* genome (version 6.2) were performed using STAR (version 2.5)^29^. The read counts from each sample were generated using HTSeq (version 0.6.0.1)^30^ with mode 'union'. Duplicate reads were found using picard tools (version 1.141). Generation of MDS plots were generated using plotMDS function from edgeR package (version 3.12.1) to visualise the level of similarity of individual cases of a dataset. From the 14 RNA-seq samples, two samples N27C23TR+ and N08C8TR+ were identified as outliers in MDS plots of RNA-seq data therefore they were excluded for final analysis (Fig. S1)^28^.

### Differential expression analysis

‘TR − ve cats’ are defined as teeth collected from a cat without any TR lesion and ‘TR + ve cats’ are teeth collected from a cat with at least one TR lesion. ‘TR tooth’ compares TR status of individual teeth, hence TR − ve teeth were collected from both TR free cats and TR diagnosed cats as long as the tooth was free of TR when assessed on dental radiographs. ‘Paired TR’ compares TR − ve teeth and TR + ve teeth from the same individuals, so a ‘within cat’ comparison.

From the remaining 12 libraries, three comparisons were performed: (1) TR − ve cats (n = 3) and TR + ve cats (n = 5), (2) TR − ve teeth (n = 7) and TR + ve teeth (n = 5), and (3) paired TR, TR − ve (n = 3) and TR + ve (n = 3). Differentially expressed (DE) genes were identified through comparisons between TR − ve and TR + ve groups using the R package edgeR (version 3.12.0)^31^. The trimmed mean of M-values normalization method was used. To explore sample dispersion between TR − ve and TR + ve groups, distance plot of biological coefficient of variation samples were generated and outliers were excluded for DE gene analysis. Significantly DE genes were identified using the threshold of false discovery rate (FDR)-adjusted *p* value < 0.05.

### Enrichment analyses

We performed two enrichment analyses on the up- and downregulated DE genes: of KEGG (Kyoto Encyclopaedia of Genes and Genomes) metabolic pathways^32^ and GO (Gene Ontology) terms^33^. GO term enrichment was assessed using the R/Bioconductor package topGO v2.38.1^34^. KEGG pathway enrichment was performed using the R/Bioconductor package SPIA (Signalling Pathway Impact Analysis) v2.38.0^35^.

### Quantitative PCR

Real-time PCR was performed using the Takyon Low ROX SYBR 2X MasterMix kit (Eurogentec) in the Stratagene MX3000P qPCR system (Agilent Technologies). Each reaction contained 300 nM of gene of interest or reference gene primer, 2 × MasterMix, 4 μl of diluted cDNA and nuclease free water, producing a final volume of 20 μl in a 96 well plate format. All samples were performed in duplicate and cycling threshold (Ct) values for each gene were generated. Calculation of relative gene expression analysis was based on delta-delta method^36,37^. The primers used are described in Table S1.

### Generation of feline osteoclasts in vitro

The protocol was modified from a published rodent osteoclast culture protocol^38^. The long bones were dissected from cats at post mortem and cut off at the proximal and distal ends. Bone marrow was flushed out in α-MEM containing 10% FBS followed by red cell lysis in Lysis Buffer (150 mM NH_4_Cl, 10 mM NaHCO_3_, 0.1 mM EDTA) for 5 min, on ice. The bone marrow cells were resuspended in α-MEM containing 10% FBS, 2 mM L glutamine, 100 IU/ml benzyl penicillin, 100 mg/ml streptomycin, and CSF-1 (recombinant mouse CSF-1, 10 ng/ml, R&D Systems) and plated on a T75 flask for 24 h at 37 °C in 5% CO_2_/95% air. On the following day, non-adherent cells were collected and plated on a dentine disc in each well of a 24 well plate (dentine discs were a generous gift from Professor Timothy R. Arnett, University College London, London, UK) or Corning Osteo Assay Surface multiple well plates (1 × 10^5^ cells/cm^2^) with CSF-1 (10 ng/ml) at 37 °C in 5% CO_2_/95% air. On day 4, 90% of the medium was changed and, replaced with a new α MEM containing CSF-1 (10 ng/ml) and RANKL (recombinant mouse RANKL, 3 ng/ml, R&D Systems). The culture media was subsequently changed twice weekly for up to 10–14 days.

### Characterisation of cultured osteoclast culture

Cultured osteoclasts on a cell culture plastic or dentine discs were washed twice with phosphate buffered saline (PBS) and fixed in 2.5% glutaraldehyde for 5 min. Tartrate resistant acid phosphatase (TRAP) activity was determined using Acid Phosphatase, Leukocyte (TRAP) Kit (Sigma-Aldrich) according to the manufacturer’s protocol with some modifications including reduced incubation time (30 min) and without haematoxylin counter staining. TRAP positive cells were imaged via light microscopy (Leica DM LB2, Milton Keynes, UK). Cells were considered to be osteoclast-like when TRAP positive and with more than two nuclei when manually counted. Resorption pits in dentine discs were visualised and counted under light microscopy after removal of cells by sonication for 5 min in 0.25 M NH_4_OH followed by 2 min toluidine blue staining.

### Inhibition of MMP9 in osteoclastogenesis

The MMP9 inhibitor containing a hydroxamate (-CONHOH) group was purchased from Calbiochem (Merck Millipore, UK) and was reconstituted in DMSO (1 mg/ml stock solution) and stored at − 20 °C. Primary feline osteoclasts derived from bone marrow were treated with a range of concentrations from 5 nM to 1 µM diluted in α-MEM until termination of osteoclast differentiation. Vehicle controls were treated with DMSO concentrations corresponding to the highest drug concentration used in the experiment. Untreated control cells were also included in each experiment.

### Targeting of siRNA of MMP9

The siRNA sequence against the feline *MMP9* was designed using an online design tool, i-Score designer (i-score web service program). *MMP9* feline siRNA targeting the *MMP9* mRNA sequence (CCAGGAGACTTGCGAACTA) was constructed using the Silencer siRNA Construction Kit (Ambion) according to the manufacturer’s protocol. Osteoclast precursors were incubated for two days in α-MEM containing 10% FBS, 2 mM L glutamine, 100 IU/ml benzyl penicillin, 100 mg/ml streptomycin, and CSF-1 (10 ng/ml) at 37 °C/5% CO_2_. Cells were detached and resuspended for electroporation in 100 μl Mouse Macrophage Nucleofector solution and siRNA. The cell suspension–DNA mixture was transferred to Amaxa electrode cuvettes and electroporated in an Amaxa Nucleofector II (Lonza, UK) using program Y-001. Following electroporation, cells were immediately suspended in medium containing CSF1 (10 ng/ml) and RANKL (3 ng/ml) and seeded into 96-well plates at 1 × 10^5^ cells/well or onto dentine discs. Cells were incubated at 37 °C/5% CO_2_ until osteoclast formation was observed in control wells. Media were changed every other day.

### Immunohistochemistry of feline teeth

Feline tooth samples were collected and fixed by immersion in 10% neutral buffered formalin for 48 h before being processed. All fixed teeth were demineralised in EDTA pH 7.0 for 4–6 weeks at room temperature (RT). Demineralised samples were dehydrated and embedded in paraffin wax following standard procedures. Haematoxylin and Eosin (H & E) staining was performed using an autostainer (Leica Autostainer XL). For MMP9 immunostaining, slides were dewaxed and rehydrated. Antigen retrieval was performed in 0.1 M citrate buffer pH 6.0 for 90 min at 70 °C. Endogenous peroxidase was blocked using 1% hydrogen peroxidase in methanol for 30 min. A further blocking was carried out using a normal goat serum 1:5 in 5% FBS for 30 min. Primary MMP9 antibody (rabbit polyclonal; Abnova, PAB12714) was diluted 1:200 in 5% of FBS in PBS and incubated at 4 °C overnight. Sections were washed and horseradish peroxidase labelled secondary antibodies (Envision Kit, Dako, UK) were added to the sections and incubated for 1 h at RT. Sections were washed 3 times with PBS for 5 min. Labelling was developed using ImmPACT DAB (Vector Labs, UK) at RT for 10 min producing a brown colour in positive sections and was counter-stained with haematoxylin.

### Statistical analysis

All experiments were repeated at least twice on two separate occasions gaining similar results (except RNA-seq). In vitro experiments included triple to five technical replicates. Quantitative experiments were analysed using Minitab 15 Statistical Software (Minitab Ltd., UK) and all graphs and diagrams were generated using Microsoft Office 2013 (Microsoft Corporation) or GraphPad prism v5 software. *P* values of less than 0.05 were deemed statistically significant. The two sample t-test or Mann Whitney U-tests were used to compare differences between two groups.

#### Results

### RNA seq data of TR − ve and TR + ve teeth from the same cat (paired comparison) had the largest number of significantly DE genes

**Figure 1:**
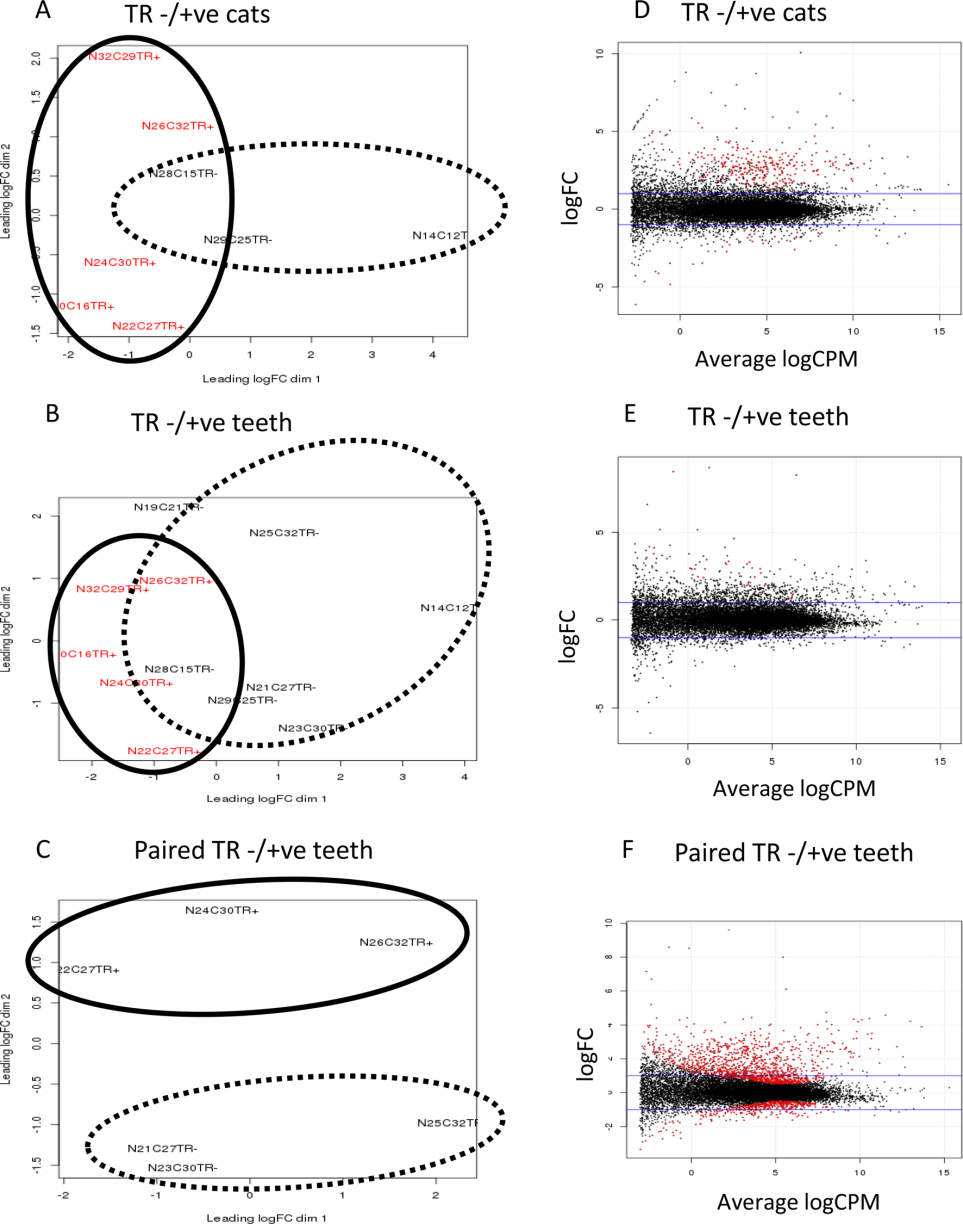
MDS plot and visualisation of gene expression data by smearplots. MDS plots of (**A**) TR −/+ cats comparison, (**B**) TR −/+ teeth comparison, and (**C**) paired TR −/+ comparison. Smear plots of (**D**) TR −/+ cats comparison, (**E**) TR −/+ teeth comparison, and (**F**) paired TR −/+ comparison. Lined circle: TR + ve samples, dotted circle: TR − ve (control teeth). Horizontal blue lines indicate fold-change of two. Red dot indicates each differentially expressed gene at False Discovery Rate or corrected *p* value (FDR) of 0.05 or smaller.

To explore the overall expression profiles between samples, multidimensional scaling (MDS) plotting was used. MDS plot of TR − ve and TR + ve samples in both TR −/+ ve cats and TR −/+ ve teeth comparisons were closely plotted which implied their expression levels were similar (Fig. 1A,B). On the contrary, in the paired comparison, TR − ve samples were widely separated from TR + ve samples indicating possession of more DE genes than the other two comparisons (Fig. 1C). The smear plot allowed for visualisation of the relationship between overall expression level measured in counts per million (CPM) on the x axis and log2 fold-change (FC) on the y axis where DE genes are displayed in red (Fig. 1D–F). The largest number of DE genes was observed in paired TR −/+ ve teeth, with 1,286 up-regulated genes and 466 down-regulated genes identified (Table 2). All expressed genes and significantly differentially expressed genes are listed in Table S2. Additionally, twenty seven differentially expressed genes were common for all three comparisons, including 25 up-regulated genes and 2 down-regulated genes (Table 3).

**Table 2:** Summary of differentially expressed genes from all comparisons.

Comparison	Total number expressed genes	Number significantly DE genes (FDR < 0.05)	Number upregulated genes	Number downregulated genes
TR- versus TR+ cats	13,243	315	279	36
TR- versus TR+ teeth	13,204	44	41	3
Paired TR- versuss TR +	13,121	1,732	1,286	446

DE: differentially expressed, FDR: false discovery rate as adjusted p-value. Total number of expressed genes (Log2 counts per million > 1).

**Table 3:** Common genes from all three comparisons.

Gene	Description	LogFC	LogCPM	Gene	Description	LogFC	LogCPM
**Upregulated genes**							
*CA6*	Carbonic anhydrase VI	10.45	2.23	*MUCIN7*	Mucin 7, secreted	8.00	5.43
*ARX*	Aristaless related homeobox	6.70	2.34	*CHGB*	Chromogranin B	4.14	1.99
*HOXA4*	Homeobox A4	3.79	2.07	*TECRL*	Trans-2,3-enoyl-CoA reductase-like	3.55	2.20
*PPP1R27*	Protein phosphatase 1 regulatory subunit 27	3.38	4.11	*LBX1*	Ladybird homeobox 1	3.12	2.10
*METTL7B*	Methyltransferase like 7B	2.71	1.00	*ATP1A3*	ATPase, Na + /K + transporting, alpha 3 polypeptide	2.54	2.22
*CA4*	Carbonic anhydrase IV	2.53	2.73	*ETNPPL*	Ethanolamine-phosphate phospho-lyase	2.52	3.34
*SLC9A2*	Solute carrier family 9, subfamily A (NHE2, cation proton antiporter 2), member 2	2.41	3.48	*CLDN10*	Claudin 10	2.35	5.31
*SMCO1*	Single-pass membrane protein with coiled-coil domains 1	2.24	2.20	*FAM166B*	Family with sequence similarity 166 member B	1.97	1.12
*LDHD*	Lactate dehydrogenase D	1.96	4.37	*TLX1*	T-cell leukaemia homeobox 1	1.91	1.28
*GRB14*	Growth factor receptor bound protein 14	1.62	2.10	*CHCHD10*	Coiled-coil-helix-coiled-coil-helix domain containing 10	1.58	5.84
*ATP2A2*	Sarcoplasmic/endoplasmic reticulum calcium ATPase 2	1.48	8.05	*CES2*	Carboxylesterase 2	1.21	3.69
*IVD*	Isovaleryl-CoA dehydrogenase	1.08	5.86	*KIF19*	Kinesin family member 19	1.05	3.55
**Downregulated gene**							
*SOSTDC1*	Sclerostin domain containing 1	− 1.05	4.06	*KLK5*	Kallikrein related peptidase 5	− 1.19	4.80

LogFC; log2 fold change, LogCPM; log2 counts per million. Upregulated genes have log2 fold change > 0 and downregulated genes have log2 fold change < 0.

### Pathway analysis revealed up-regulated genes that are involved in osteoclast differentiation and calcium signalling

**Figure 2:**
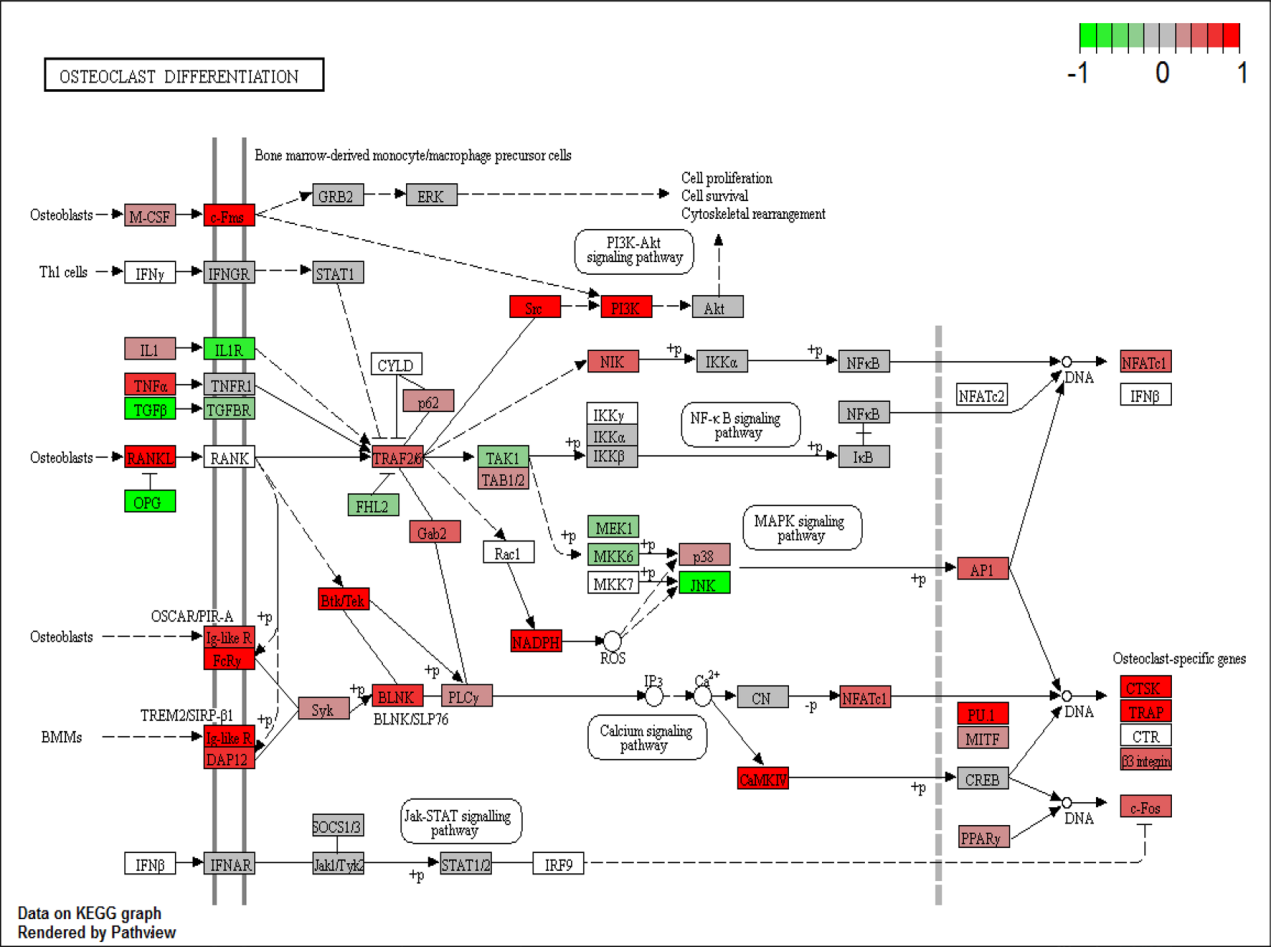
Osteoclast differentiation pathway (map04380) obtained from KEGG database^32^ with official permission and guidance from Kanehisha Laboratories (permission ref 200,290). Expressed genes are coloured with red indicating relative up-regulation and with green indicating down-regulation in TR + ve teeth.

**Figure 3:**
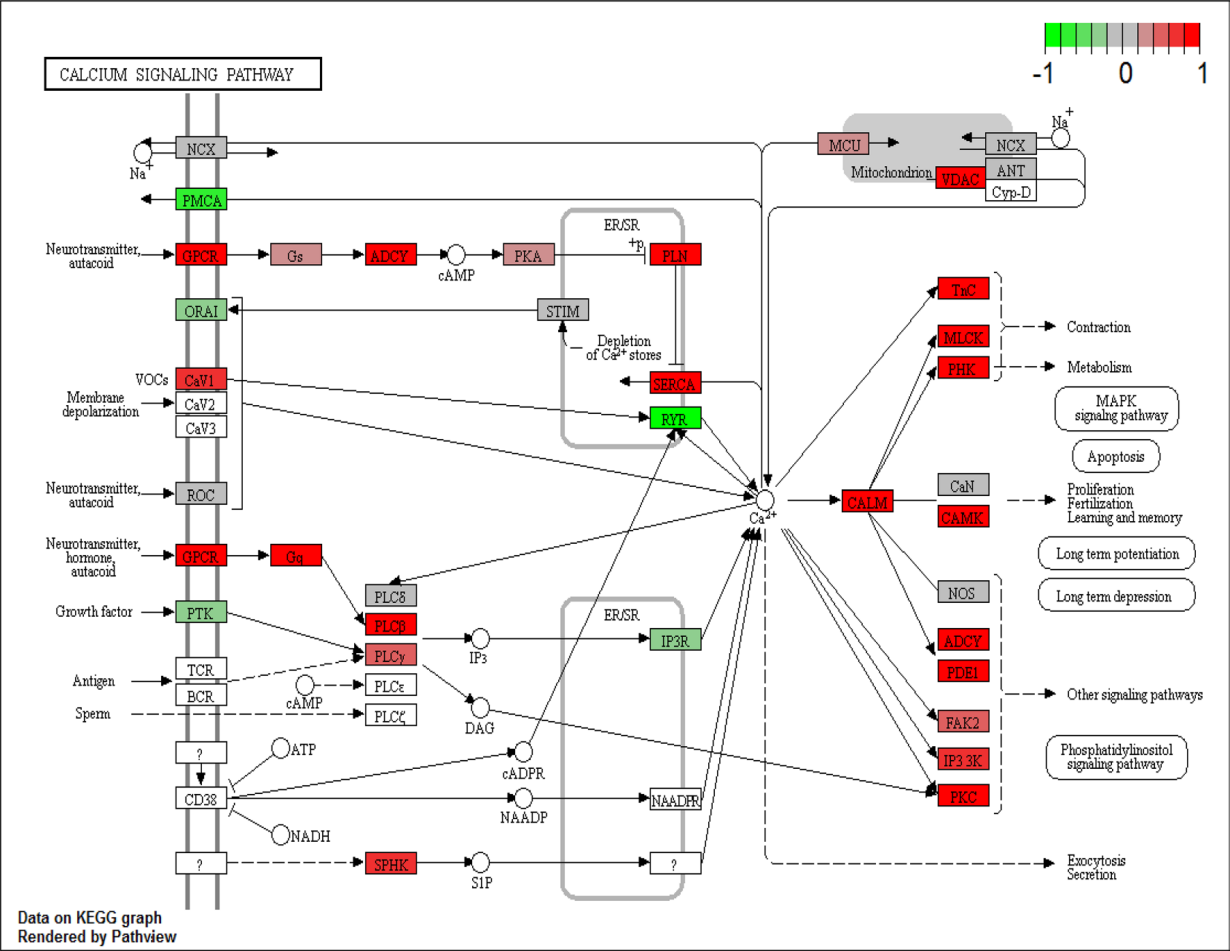
Calcium signalling pathway (map04020) obtained from KEGG database^32^ with official permission and guidance from Kanehisha Laboratories (permission ref 200,290). Expressed genes are coloured with red indicating relative up-regulation and with green indicating down-regulation in TR + ve teeth.

For further characterisation of the transcriptomic changes involved in TR, enrichment analyses were performed on the set of 1,732 genes DE between paired TR groups. KEGG downstream pathway analysis identified a total of 28 metabolic pathways within which these genes were involved (Table S3). Of these, the set of upregulated genes were enriched among the genes comprising 25 pathways, with the set of downregulated genes enriched only among 3 pathways. The most biologically relevant pathways were osteoclast differentiation (Fig. 2 and Table S3, KEGG pathway 04380; *p* = 0.03) and calcium signalling (Fig. 3 and Table S3, KEGG pathway 04020; *p* = 1.1 × 10^–4^) which promote the differentiation of osteoclast precursors and induce osteoclastic tooth resorption in teeth. Relative expression of each gene in each pathway was visualised with colour labels in Figs. 2 and 3. Identified pathways and their active status were revealed based on the expression of genes which constitute that pathway (Table S3). For instance, key genes in the osteoclast differentiation pathway were highly expressed in TR + ve teeth (Table S3, total number of genes in osteoclast differentiation pathway = 93, number of up-regulated differentially expressed genes = 15, down-regulated differentially expressed genes = 3). RANK is significantly up-regulated (Table 4, log2 fold change = 1.157, FDR = 0.0013) in TR + ve teeth. RANKL was not significantly up-regulated (Table S2, log2 fold change = 0.810, FDR = 0.253) but OPG was down-regulated (Table 4, log2 fold change = −1.326, FDR = 7.0 × 10^–8^), which led to an overall increase in the RANKL/OPG ratio. Table 4 highlighted differentially expressed genes in osteoclast differentiation and calcium pathway with putative roles. Gene ontology enrichment analysis presented many gene sets involved in muscle physiology including actin binding, myofibril, actin cytoskeleton, muscle contraction, heart contraction, muscle fibre development, skeletal muscle cell differentiation and muscle organ morphogenesis (Table S4).

**Table 4:** DE genes paired comparison. LogFC; log2 fold change, FDR: false discovery rate as adjusted p-value.

Gene status	Gene name	Description	logFC	FDR	Putative function
**Putative pathway: osteoclast differentiation**					
↑	*SPI1* *(PU.1)*	Spi-1 proto-oncogene	1.028	0.0014	Induction of osteoclast formation^39^
↑	*C-FMS* *(CSF1R)*	colony stimulating factor 1 receptor	1.026	1.37 × 10^–6^	A trigger event leading to osteoclast differentiation via the c-Fms receptor^40^
↑	*TNFRSF11A (RANK)*	TNF receptor superfamily member 11a	1.157	0.0013	RANK/RANKL signalling is a central regulator of osteoclastogenesis^41^
↑	*TNFSF11* *(RANKL)*	TNF superfamily member 11	0.810	^#^0.253	
↑	*OCSTAMP*	osteoclast stimulatory transmembrane protein	2.329	0.0030	Lack of cell–cell fusion of osteoclasts in OC-STAMP deficient mice^42^
↑	*ACP5*	acid phosphatase 5, tartrate resistant	2.149	0.007	A cytochemical marker of osteoclast^43^
↑	*MMP9*	matrix metallopeptidase 9	2.326	0.010	High expression in RANKL induced osteoclasts^44^
↑	*CALCR*	calcitonin receptor	1.896	0.036	Increase of calcitonin receptor during osteoclast formation^45^
↑	*CTSK*	cathepsin K	1.099	*0.059	Degradation of type I collagen by osteoclast-mediated bone resorption^46^
↑	*TREM2*	triggering receptor expressed on myeloid cells 2	1.293	0.0027	Co-stimulatory receptor of RANK signalling^47^
↑	*TYROBP* *(DAP12)*	TYRO protein tyrosine kinase binding protein	0.764	0.0011	Osteopenia due to increase of osteoclasts in DAP12 overexpression mice^48^
↑	*CYBA* *(Nox1)*	cytochrome b-245 alpha chain	0.639	0.0080	MAPK activation via Nox1 in osteoclast differentiation^49^
↑	*CYBB* *(Nox2)*	cytochrome b-245 beta chain	1.014	0.0146	Increase of Nox2 during RANKL induced osteoclasts^49^
↓	*TNFRSF11B* *(OPG)*	TNF receptor superfamily member 11b	− 1.326	7.0 × 10^–8^	A negative regulator of osteoclastogenesis^41^
↓	*TGFB1*	transforming growth factor beta 1	− 0.626	0.024	Inhibitory effect of TGF-β on osteoclast differentiation^50^
↓	*TGFB2*	transforming growth factor beta 2	− 0.697	0.0055	
**Putative pathway: calcium signalling**					
↑	*P2X2R*	purinergic receptor P2X2	1.962	4.3 × 10^–4^	Induction of Ca^2^^+^ influx in osteoclast precursors and osteoclasts via extracellular ATP via various purinergic receptors^51–53^
↑	*P2X4R*	purinergic receptor P2X4	0.786	0.0029	
↑	*P2X6R*	purinergic receptor P2X 6	2.700	1.26 × 10^–5^	
↑	*PLCB4*	phospholipase C beta 4	1.106	3.36 × 10^–5^	Activated by GPLC^52,53^
↓	*P2X7R*	purinergic receptor P2X7	− 0.271	^#^0.639	Increased bone resorption by loss of function of P2 × 7r^54^

### RT-qPCR validated the differential expression of the selected genes from RNA-seq result

Twelve candidate genes were functionally associated with osteoclast differentiation and activity (Tables 3 and 4) and were selected for validation by qPCR (Table 5). All genes considered significantly DE by RNA-seq (*SPI1, MMP9, OPG, RANKL/OPG, ACP5, P2X2R, P2X4R, P2X6R, PCLB4, CA4* and *CA6*) were confirmed to be up-regulated in TR + ve teeth by qPCR. However, 3 genes considered DE by RNA-seq (*CA4, P2X6R,* and *PCLB4*) could not be confirmed by qPCR (Table 5). *RANKL* expression was unchanged but the gene expression ratio of *RANKL/OPG* was higher in TR + ve teeth (Fold change = 3.10, *p* = 0.036) compared to TR –ve teeth. *CA6* was identified as the most highly upregulated gene in TR + ve teeth (Fold change = 5.51), followed by *MMP9* (Fold change = 4.52), *P2X2R* (Fold change = 3.04), *ACP5* (Fold change = 2.95), *SPI1* (Fold change = 2.28) and *P2X4R* (Fold change = 2.28). *CTSK* was highly upregulated in TR + ve teeth by qPCR (*p* = 0.003) but not by RNA-seq (FDR = 0.059). Overall the gene expression profile of TR identified by RNA-seq was largely consistent with that of the qPCR analysis. Differential expression of *MMP9* and *P2X4R* were re-confirmed by qPCR on a further six paired TR − ve and TR + ve teeth samples although this was not the case for *SPI1, RANKL, OPG* and *RANKL/OPG* (Fig. S2).

**Table 5:** RNA-seq result validation by qPCR.

Putative function	Gene	RNA-seq	qPCR		
		Expression in TR+ teeth	Expression in TR+	Fold change normalised to TR-	*p* value
Osteoclast differentiation	*SPI1*	↑**	↑**	2.60	0.024
	*RANKL*	↑	↑	1.35	0.279
	*OPG*	↓**	↓**	0.44	0.037
	*RANKL/OPG*	–	↑**	3.10	0.036
Osteoclast genes	*MMP9*	↑**	↑**	4.52	0.016
	*ACP5*	↑**	↑**	2.95	0.019
	*CTSK*	↑*	↑**	2.81	0.003
Calcium signalling in osteoclast	*P2X2R*	↑**	↑**	3.04	0.025
	*P2X4R*	↑**	↑**	2.28	0.039
	*P2X6R*	↑**	↑*	2.81	0.079
	*PCLB4*	↑**	↑	1.83	0.100
Common genes in TR + teeth (pH homeostasis)	*CA4*	↑**	↑*	2.67	0.052
	*CA6*	↑**	↑**	5.51	0.040

Total n = 6 from TR –ve = 3, from TR + ve = 3, *; 0.05 < *p* < 0.1, **; *p* < 0.05 by student t-test.

### MMP9 is highly expressed in odontoclasts present in feline tooth resorption pits

**Figure 4:**
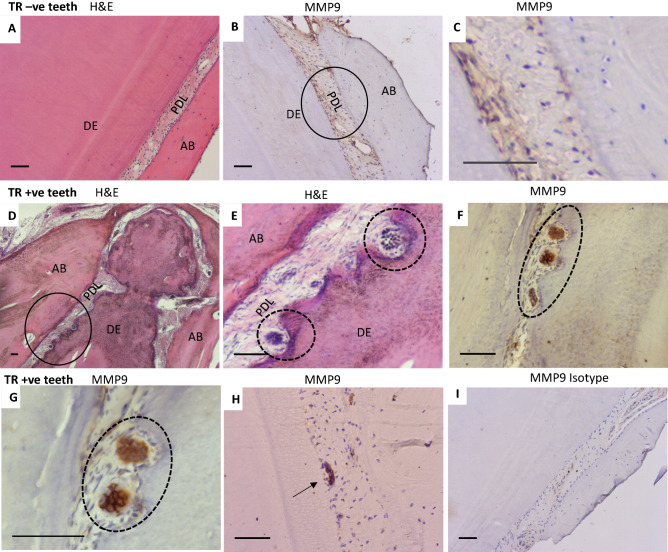
MMP9 is highly expressed in odontoclasts of feline tooth resorption lesions. H&E from TR − ve teeth (**A**) and TR + ve teeth (**D**,**E**). Immunohistochemical labelling for MMP9 protein with haematoxylin counterstaining TR − ve (**B**,**C**), TR + ve sections (**F**,**G**,**H**) and isotype control (**I**). No visible odontoclast is found in TR − ve teeth (**B**) and circle area of **B** is magnified in **C**. Resorbing odontoclasts (**D**, circle area) are magnified in **E**. MMP9 expression in TR − ve teeth are observed mainly in periodontal ligament or gingival tissues (**B**,**C**). TR + ve teeth contain active odontoclasts with resorption pits (**F**, dotted circle) with strong MMP9 expression, high magnification of this lesion (**G**). MMP9 expression was also found in multinucleated like cells but these cells did not reside in a resorption pit (**H**, arrow). *AB* alveolar bone, *DE* dentine, *PDL* periodontal ligament. Scale bars = 100 µm.

MMP9 was successfully localised in feline dental tissues by immunohistochemistry (Fig. 4). Multinucleated odontoclast-like cells based on H&E staining (Fig. 4D,E) and MMP9 immunolabelled odontoclasts were identifiable in TR + ve sections (Fig. 4E,F) and there was no odontoclasts observed in TR − ve sections (Fig. 4A,B). Quantitative analysis was not carried out due to the limited number of sections and number of active odontoclasts. Since aggregated gingival epithelium also expressed some MMP9 (Fig. 4H), only multinucleated cells within an obvious resorption pit were considered to be odontoclasts. High expression of MMP9 was observed in actively resorbing odontoclasts in TR lesions (Fig. 4F,G). Fibroblasts of the periodontal ligament also expressed MMP9 in both TR − ve (Fig. 4B,C) and TR + ve teeth (Fig. 4F,G). It was also noted that the majority of TR + ve teeth had disrupted periodontal ligament fibres, narrowed or complete loss of periodontal ligament space and replacement with bone like tissues demonstrating a degree of ankylosis.

**Figure 5:**
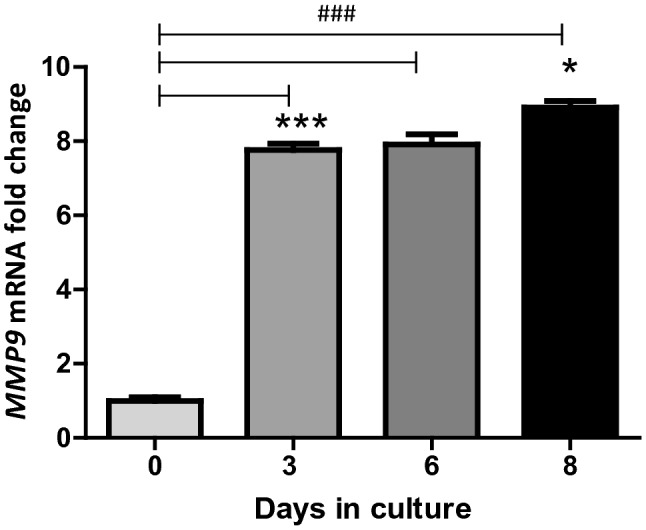
*MMP9* mRNA expression increased during in vitro feline osteoclast formation over an 8 day culture period. Precursors were treated with M-CSF from day 0, and RANKL was added from day 3. Graphs represent relative expression as fold changes with bars showing standard error of the mean. (n = 3; *p < 0.05, ***p < 0.001 in comparison with the previous time point in culture, ^###^*p* < 0.001 in comparison with day 0 by two sample *t* test).

*MMP9* expression was confirmed in cultured feline osteoclasts and increased upon osteoclast formation. *MMP9* mRNA expression was significantly up-regulated in the early stages of osteoclast differentiation, with a 7.8 ± 0.2 (*p* < 0.001) fold change on day 3 compared to day 0. *MMP9* mRNA expression remained high until day 6 where after it increased further 8.9 ± 0.2 (*p* = 0.0387) fold change on day 8 (Fig. 5).

### Treatment with a semi-selective MMP9 inhibitor reduced osteoclast differentiation and resorption activity

**Figure 6:**
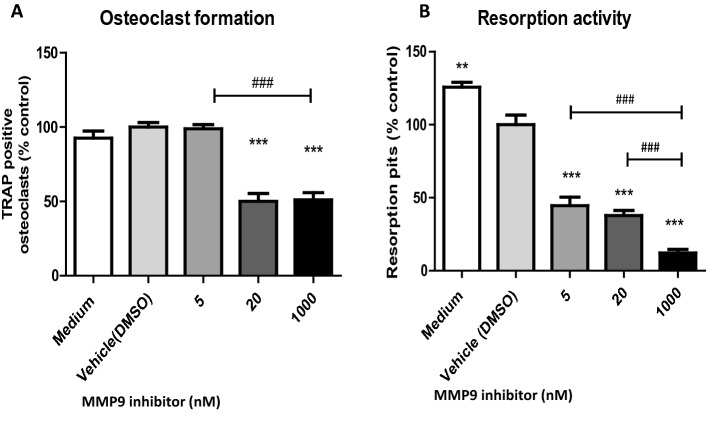
MMP9 semi-selective inhibitor decreased feline osteoclast formation and resorption activity. Graphs represent percentage of number of osteoclasts (**A**) or resorption pits (**B**) with + SEM bars compared to vehicle (DMSO) control. (n = 3; *p < 0.05, **p < 0.01; ***p < 0.001 in comparison with vehicle control, ^###^*p* < 0.001 between individual doses comparison by two sample *t* test).

In vitro osteoclast cultures were treated with a semi-selective MMP9 inhibitor (hydroxamate-based MMP inhibitor). Inhibitory effects on osteoclast formation and resorption activity were observed in a dose dependent manner (Fig. 6). Osteoclast formation was not significantly reduced at low dose (5 nM, the half maximal inhibitory concentration = 5 nM) compared to vehicle control (*p* = 0.802), but the inhibitor significantly prevented osteoclast formation at higher doses, whereby 50 ± 6.3% (*p* < 0.0001) and 48.9 ± 5.8% (*p* < 0.0001) of the number of osteoclasts formed were observed compare to vehicle at 20 nM and 1000 nM treatments, respectively (Fig. 6A). There was no difference in osteoclast numbers when increasing the concentration from 20 to 1000 nM (*p* = 0.888). All three concentrations of MMP9 inhibitor resulted in reduction of resorption pits compared to vehicle control (Fig. 6B). This inhibitory effect was dose dependent with 5, 20 and 1000 nM treatments producing 62.2 ± 7.4% (*p* = 0.002), 55.4 ± 8.8% (*p* = 0.0003) and 12.2 ± 2.5% (*p* = 0.001) of the number of resorption pits observed in the vehicle control, respectively.

### *MMP9* specific siRNAs causes a moderate reduction in mRNA levels and reduced the number of osteoclasts formed

**Figure 7:**
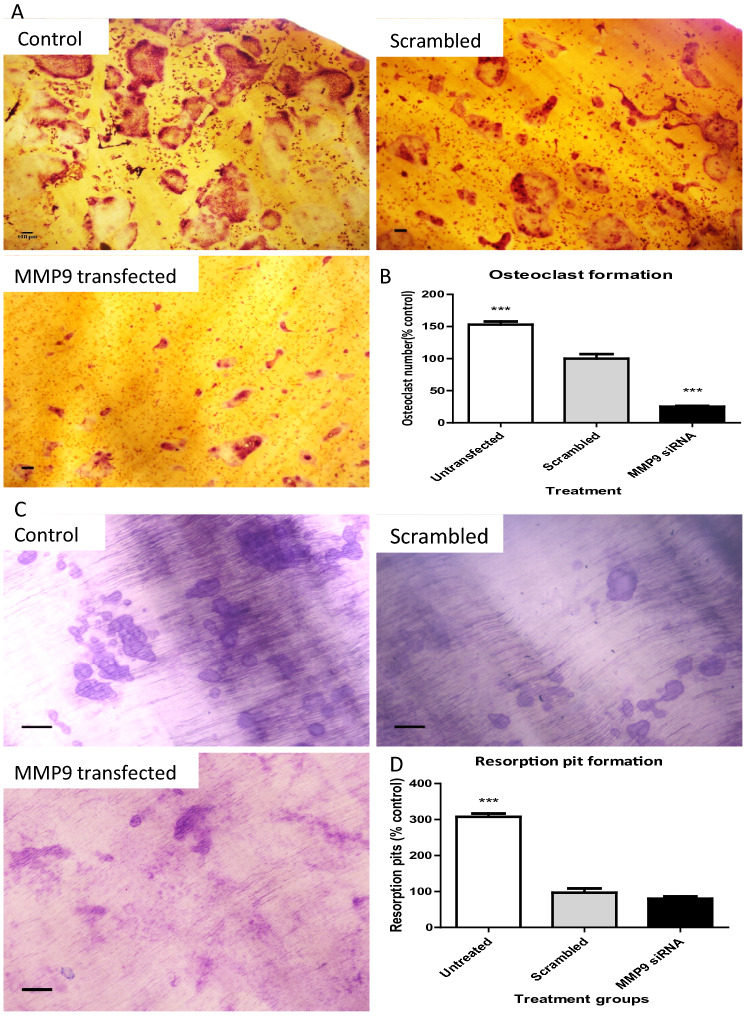
*MMP9* siRNA transfected cells resulted in reduced osteoclast formation in comparison with scrambled control but there was no statistically significant difference in resorption activity. Representative images of TRAP positive osteoclasts on dentin discs from untransfected control, scrambled control and *MMP9* siRNA transfected treatment (**A**). Representative images of toluidine blue stained resorption pits from untransfected control, scrambled control and *MMP9* siRNA transfected treatment (**C**). Graphs represent percentage of number of osteoclasts (**B**) and resorption pits (**D**) with + SEM bars. (n = 3; ***p < 0.001 in comparison with scrambled control). Scale bars = 100 µm.

Feline osteoclast precursors were transfected with feline *MMP9* siRNA or a scrambled siRNA as control and seeded on dentine discs to investigate the role of MMP9 during osteoclast formation. Following electroporation of the osteoclast precursors with the *MMP9* siRNA, a 44.00 ± 0.03% reduction (*p* = 0.0032) in *MMP9* mRNA levels was observed in the transfected cells as assessed by qPCR at 48 h post transfection when compared to negative control transfected precursors (Fig. S3). Electroporation caused some cell death and therefore untransfected cells formed a higher number of osteoclasts and more resorption pits (Fig. 7B,D) but all groups of transfected cells were also able to differentiate into mature osteoclasts. Osteoclast formation was reduced (74.8 ± 7.2%; *p* < 0.0001) (Fig. 7A,B) in *MMP9* siRNA transfected cells in comparison to scrambled siRNA control cells. However, no statistically significant differences in resorption activity was observed between scrambled siRNA and *MMP9* siRNA treated wells (*p* < 0.2298) (Fig. 7).

## Discussion

Current understanding of the aetiologies of feline TR is incomplete. Dysregulation of odontoclasts are responsible for TR but the mechanisms underlying their dysregulation are largely unknown. The central aims of this study were to investigate the transcriptomic profile of TR affected cats, identify candidate genes related to feline odontoclast dysregulation and assess their potential as therapeutic targets.

First, we performed a RNA-seq to study the whole dental transcriptome of feline teeth and identify differentially expressed genes correlated with progression of TR. No previous RNA-seq analysis of feline TR has been reported, but there are several dental transcriptomic studies using RNA-seq on rodent developing teeth and microarray studies on human teeth that suggested that different cells and tissues expressed unique transcriptomic profiles (e.g. between odontoblasts and pre-secretory ameloblasts or deciduous and permanent periodontal ligament and pulp)^55–57^.

In this study, we hypothesised that the transcriptome of TR affected teeth is locally different from TR free teeth and compared transcriptomic changes between TR − ve and TR + ve samples. There were only a few changes in gene expression that were common between all three comparisons of TR −/+ ve teeth, TR −/+ ve cat and paired TR − ve and TR + ve teeth and this may be due to large individual variations in the samples available. There were only limited genes identified in common between TR − ve and TR + ve teeth. The majority of remaining genes were uncharacterised or were not reported to be involved in tooth or osteoclast biology. Of these, carbonic anhydrase 4 (CA4) has been reported to be expressed in the plasma membrane of osteoclasts^58^ and CA6 is known to be abundantly present in salivary glands and saliva and involved in maintenance of pH homeostasis in oral cavity^59,60^. An oral microenvironment high in carbonic anhydrase may favour osteoclast differentiation^58^. Some homeobox genes (e.g. *ARX, HOXA4*, *LBX1, TLX1*) has been reported in tooth development^61^. One of the downregulated genes was *SOSTDC1* which has been reported to be largely related to tooth development as an inhibitor of Wnt signalling to control tooth number and morphogenesis^62,63^. The role of these genes in TR + ve teeth are unclear but may suggest that permanent TR + ve teeth may undergo some form of remodelling process^61,64^.

The paired TR comparison gave the most interesting results; a large number of transcripts from the TR + ve teeth differed from TR − ve teeth (n = 1732) which implies that these transcriptomic changes occur locally within the same dentition. The fact that there was transcriptomic similarity between teeth of the same phenotype and that the different phenotypes could clearly be separated reveals that the TR affected teeth undergo similar cellular and molecular changes. The transcriptome corresponding to the phenotype may suggest that transcriptomic changes are at the core of TR pathogenesis, and this knowledge has the potential to increase our understanding of the aetiology of TR.

Previous feline TR studies suggested that the local expression of inflammatory cytokines (e.g. IL1B, IL6, TNF and IFNG) and RANKL may be involved in feline TR, however expression of both pro-inflammatory and inhibitory-inflammatory cytokines increased in TR teeth than control by qPCR which makes it difficult to conclude and the local level of actual cytokines (protein) has however never been measured in TR^19,20^. Therefore overall influence of inflammation cannot be conclusively determined. In human root resorption, P2X7R was considered a stimulator of osteoclast differentiation^65^.

In this study, both RNA-seq data and qPCR result could not identify any significant changes in the expression of inflammatory cytokine genes between TR –ve and TR + ve teeth (e.g. *IL1B, IL6, TNF, IFNG)*, *P2X7R* or *VDR*, Fig. S4). It is possible that inflammation only appears at a particular stage of TR development so that the overall oral environment possesses a similar extent of inflammation within this study population. It has been reported that the prevalence of TR increased with age in random mixed breed cats although TR occurred regardless of age in pure breed cats^66,67^. In our study population, the age range was broad (from 4 to 16 years), however the mean age (7.2 years) was similar to those in the previous studies (6.2 and 6.5 years)^66,68^. To avoid bias due to age, young cats were excluded (< 3 years) in our study and there was no statistically difference of the mean age between TR − ve (7.6 ± 3.2) and TR + ve cats (6.7 ± 1.9) (*p* = 0.68).

Another possible explanation for the lack of any changes in the expression levels of inflammatory cytokines could be due to a bias of TR type in this study. In this study, type 2 was the dominant type of TR (Table 1). It has been proposed that this type might be involved in non-inflammatory or idiopathic TR although other studies have not confirmed this observation^11,69,70^. The finding that there is no change in *RANKL* mRNA levels was consistent with previous feline TR studies^19,20^. It has been reported that RANKL is highly expressed in the periodontal ligament of actively resorbing primary human teeth^71,72^, whereas the level of *RANKL* mRNA is very low or not expressed in human adult teeth^73^. In cats, *RANKL* mRNA levels in adult teeth and bones are similar^19^. Since odontoclast activity is tightly controlled by the RANKL/OPG ratio, the level of *OPG* and *RANKL* mRNA expression should be considered together. In healthy feline permanent teeth, the level of *OPG* mRNA is significantly higher than femoral or alveolar bone, providing evidence that the role of OPG may be a key factor, rather than RANKL, in modulating odontoclast function in feline TR^19^. This is supported by RNA-seq and qPCR data in the comparison of TR -/ + teeth from the same cat; the level of *OPG* mRNA level was low in the TR + ve teeth thus an increased RANKL/OPG ratio was observed in TR + ve teeth. However, the observation was not verified when repeated in a larger number of samples, hence the RANKL/OPG involvement in feline TR needs to be further investigated.

Based on the large number of DE genes, there were several interesting metabolic pathways identified. Specifically, DE genes were found which are reported to be involved in osteoclast differentiation (*SPI1, C-FMS, RANK, RANKL, TREM2*, *DAP12, Nox1* and *Nox2*)^39,41,48–51^ or highly expressed in functional and mature osteoclast (*OCSTAMP, ACP5, MMP9, CALCR* and *CTSK*)^42,43,45,46^. *SPI1*, the gene encoding the haematopoietic transcription factor PU.1, is essential for myeloid and B-lymphoid cell development^39^. This was reported to be the earliest molecule which binds to sites in both the c-fms promoter or intronic regulatory element (FIRE enhancer) for commitment to osteoclast lineage in mice^40,74^. The increased expression of *SPI1* and *C-FMS* found in TR + ve teeth may suggest that TR + ve teeth possess more odontoclast precursors in their environment which would be likely to form mature odontoclasts and induce tooth resorption. *ACP5* and *CTSK* are recognised osteoclast markers in bone and teeth^43,46^. Up-regulation of these genes also suggest increased odontoclast number and activity in TR teeth. MMP9 is a matrix metallopeptidase enzyme, gelatinase B and type IV collagenase. Its primary role is to degrade and remodel the extracellular matrix in many tissues including bone and teeth^75^. Increased MMP9 activity has been implicated in many diseases including osteoclastomas, Paget's disease, bone fracture repair and dental pulp inflammation^76,77^. It has also been reported that it is highly expressed in early stages of differentiating osteoclasts, mature osteoclasts as well as in odontoclasts in many species including human, mouse, rabbit and cow^78–80^. Recently accumulated evidence suggests that MMP9 have an important role in the progression of caries, specifically in the pulpal and periapical inflammation tissue destruction process. Significant elevation of MMP9 expression was observed in apical periodontitis. MMP9 is synthesized and secreted by various stimulators such as physical agents or cell cytokines (IL1B and TNF-α)^77^. Interestingly, Zhang et al. (2020) suggested that MMP9 may have anti-inflammatory properties^81^. They showed that MMP9 knockout mice experimentally induced with apical periodontitis suffered a more severe inflammation than wild type mice. It was also reported that MMP9 induced the reduction of IL1B, TNF-α, RANK, RANKL, TLR2, and TLR4 and increased OPG expression in LPS-stimulated osteoblasts. These findings suggest that the role of MMP9 in bone destruction is complex and diverse and emphasises the role of MMP9 as an inflammatory regulator by osteoblasts^81^. In our data, MMP9 expression was higher in TR + ve teeth but there was no difference in expression of inflammatory cytokines (IL1B, TNF-α) or RANK. Feline TR starts normally at the external root and neck area rather than within the pulp, and often results in ankylosis between tooth and alveolar bone rather than alveolar bone destruction. Feline TR is a unique phenomenon and might indicate slightly different pathogenesis from typical inflammation. Our study primarily focused on the role of the odontoclast, but it might be necessary to investigate further roles of other cell types comprising of the tooth (odontoblast, periodontal ligament cells). However, expression of MMP9 and its role in bone and tooth resorption has not previously been described in cats. One study reported high expression of MMP9 in feline cancer^82^.

Another pathway identified, the calcium signalling pathway, controls multiple cellular functions in many cells via release of calcium ions from internal stores and its entry from the extracellular fluid^53^. In particular, calcium signals in osteoclasts are responsible for diverse cellular functions including differentiation, bone resorption and gene transcription^52^. Genes in calcium signalling might induce Ca^2+^ influx thus contributing to osteoclast differentiation (*P2X2R, P2X4R, P2X6R*, *PLCB4*)^51–53^. These genes are known to encode for nonselective cation channels permeable to Ca^2+^ at the cellular level and thereby contribute to osteoclast differentiation. Originally, purinergic receptors were thought to play a role in ATP production which would in turn induce the formation and activation of osteoclasts^51,83^. Purinergic receptors e.g. P2X7 or P2Y have been implicated as potent local inhibitors of matrix mineralisation^83^. Expression of P2X2 receptor has been reported in both osteoblasts and osteoclasts but its precise role in bone is not clear^51^. Up-regulation of P2X4 receptor has been attributed to inflammation via macrophage invasion^84^. Although expression of the P2X6 receptor has not been reported in osteoclasts, its expression was reported to modulate the differentiation and migration of human mesenchymal stem cells^85^. The roles of these genes (and their encoded proteins) in the dental microenvironment is largely unknown, so further studies are required to investigate their possible involvement in tooth resorption.

In addition to osteoclast biology, gene ontology enrichment analysis showed abundant genes involved in muscle physiology. Genes involved in structural myofibril components including *ACTA1, ACTN2, ATP2A1, CSRP3, MYH11, MYOZ2, PDLIM3, SYNC, TCAP*, *TNNI1*, *TNNT3* and *TPM2* were all up-regulated, however, the exact role of these genes in bone and tooth structure/function is unclear. These genes have also been identified to play a role in the development and differentiation of osteocytes, mechanosensing cells that coordinate the remodelling process mediated by osteoblasts and osteoclasts in developing bone and tooth^86^. This manifestation is unexpected as adult teeth normally possess a limited ability to remodel. However, as TR + ve teeth showed a predominantly disrupted periodontal ligament, new bone formation (ankylosis) and external TR lesions in the root rather than internally, the noted changes in gene expression may reflect repair and some limited remodelling in the roots of the TR + ve teeth, possibly resulting in the level of ankylosis noted in the histological sections. Of the above DE genes, we verified RNA-seq results with qPCR for the genes *SPI1, OPG, MMP9, ACP5, CTSK, P2X2R, P2X4R, P2X6R* and *PCLB4* which all might be involved in osteoclast differentiation and activity, based on previous reports^43,46,51,74,75,87–89^.

Finally, an in vitro feline osteoclast model was established to test the potential role of candidate genes. Since our data showed *MMP9* was one of the most up-regulated genes in TR + ve teeth, it was chosen as a target for further investigation. Inhibition of MMP9 by a semi-selective synthetic inhibitor caused both a reduction of osteoclast formation and resorption activity. Synthetic MMP inhibitors have been developed for the treatment of several serious pathologies including periodontitis, although clinical trials gave disappointing results and only a few inhibitors (e.g. Periostat™) has been approved by the FDA for the treatment of periodontal disease^90^. The synthetic inhibitor possesses a hydroxamic acid analogue which binds to the enzymatic sites of MMPs with high-affinity. However, members of MMPs family share a basic structure which comprises an auto-inhibitory pro-domain rendering them enzymatic latency, the catalytic domain, and the C-terminal hemopexin-like domain, for the recognition of MMP substrates^91^. Although the inhibition of osteoclastic activity by the MMP9 inhibitor is concentration dependent, it is not clear if the effect of the MMP9 inhibitor is specific to MMP9 or not. Therefore, MMP9 inhibitor experiments were confirmed using the gene specific siRNA approach. When inhibition of *MMP9* mRNA was successfully performed by feline MMP9 siRNA, osteoclast formation was inhibited, but not resorption activity. The reduction of resorption activity by synthetic inhibitors but not by the more specific siRNA approach may be a result of off-target inhibition of other members of the MMP superfamily. These results suggest that the role for MMP9 in TR pathogenesis requires further investigation before any firm conclusions can be reached. Strong MMP9 expression both in osteoclasts and odontoclasts has been reported in bovine, rabbit and mice skeletal tissue^78,92^, and was seen here in the histological tooth sections. Therefore MMP9 may be a potential therapeutic target in feline tooth resorption. The underlying mechanism of inhibition of osteoclast formation by MMP9 needs to be further investigated. Other candidate genes verified in this study could also be potential therapeutic targets in feline TR, and further studies, potentially looking at co-targeting of multiple targets should be performed.

## Supplementary information

Supplementary Information 1.

Supplementary Information 2.

Supplementary Information 3.

Supplementary Information 4.

Supplementary Information 5.
