# Supplementary material for: Transcriptomic profiling of feline teeth highlights the role of matrix metalloproteinase 9 (MMP9) in tooth resorption
Source: Sci Rep. 2020 Nov 3;10:18958. doi: 10.1038/s41598-020-75998-3 (PMC7641192; doi:10.1038/s41598-020-75998-3)
Supplement: Supplementary file 1 — Supplementary Information 1. [file 41598_2020_75998_MOESM1_ESM.docx]

**Supplementary Information for:**

**Transcriptomic profiling of feline teeth highlights the role of matrix metalloproteinase 9 (MMP9) in tooth resorption**

Authors:

Lee, S. ^1^, Bush, S.J.^2^, Thorne, S.^3^, Mawson N^1^, Farquharson, C. ^1^, Bergkvist, G.T. ^1^

Affiliations and addresses:

1. The Royal (Dick) School of Veterinary Studies and The Roslin Institute, University of

Edinburgh, Easter Bush Campus, Midlothian, EH25 9RG, UK

2. Nuffield Department of Medicine, University of Oxford, Oxford, OX3 7LF, UK

3. DentalVets, Apex House, Alderston Field, Haldane Avenue, Haddington, EH41 3NQ

*Correspondence to or [seungmee.lee@roslin.ed.ac.uk](mailto:seungmee.lee@roslin.ed.ac.uk) or [gura.bergkvist@ed.ac.uk](mailto:gura.bergkvist@ed.ac.uk)

**Supplementary Figures**


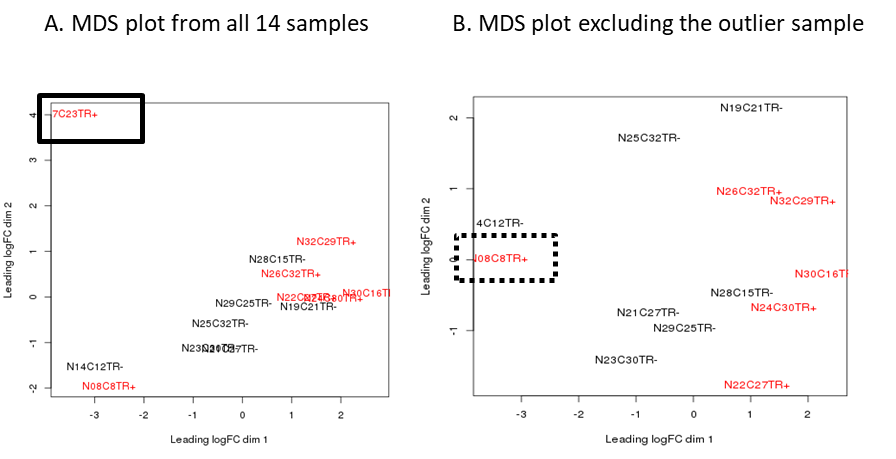


Figure S1. Sample selection for data analysis. (A) MDS plots for RNA-seq data from all 14 samples. Lined square indicates an outlier. Distance between samples indicates similarity. (B) MDS plots of RNA-seq data excluding the outlier (N27C23TR+). Dotted square, the sample N08C8TR+, shows similar data to TR -ve samples.

Figure S2. *MMP9* and *P2X4R* were highly expressed in TR -ve and TR +ve teeth. Confirmation of gene expression by qPCR in further tooth samples was performed. Graphs represent relative expression as fold changes + SEM bars. (Total n = 12, TR -ve = 6, TR +ve = 6; * *p* < 0.05, ** *p* < 0.01 by the two sample t-test).

Figure S3. *MMP9* mRNA expression levels were reduced (48 hrs post transfection) by *MMP9* siRNA electroporation of feline osteoclast precursors. Graph represents relative expression as fold changes with + SEM bars. (n = 3, ** *p* < 0.01 by two sample t-test).

Figure S4. Relative expression of inflammatory cytokines, *VDR* and *RANKL* between TR –ve and TR +ve teeth. Each gene of interest was normalised to four reference genes (*HPRT, GAPDH, RPL17, RPS19*). None of the genes showed any statistical differences in expression by the two sample t-test (P > 0.05). Graphs represent relative expression as fold changes with SEM bars (n= 11 for TR –ve teeth and n=13 for TR +ve teeth).

**Supplementary tables**

**Table S1.** List of primer sets used for quantitative PCR.

**Table S2.** All expressed and DE genes with fold change in paired comparison

**Table S3.** KEGG pathway enrichment for the set of DE genes between TR- and TR+

**Table S4.** GO term enrichment for the set of DE genes between TR- and TR+
